# Editorial: Insights in general cardiovascular medicine: 2022

**DOI:** 10.3389/fcvm.2023.1259212

**Published:** 2023-08-09

**Authors:** Maurizio Acampa, Riccardo Accioli, Viola Salvini, Junjie Xiao, Pietro Enea Lazzerini

**Affiliations:** ^1^ Stroke Unit, Department of Emergency-Urgency and Transplants, Azienda Ospedaliera Universitaria Senese, “Santa Maria alle Scotte” General-Hospital, Siena, Italy; ^2^ Department of Medical Sciences, Surgery and Neurosciences, University of Siena, Siena, Italy; ^3^ Institute of Cardiovascular Sciences, Shanghai Engineering Research Center of Organ Repair, School of Life Science, Shanghai University, Shanghai, China

**Keywords:** heart failure, atrial cardiopathy, coronary artery disease, syncope, pulmonary hypertension

**Editorial on the Research Topic** Insights in General Cardiovascular Medicine: 2022

In this Research Topic, an international selection of high-quality papers by our editorial board members contributed to highlight the latest advancements in research across the field of cardiovascular medicine. Specifically, these papers offer new insights into various conditions, including heart failure (HF), atrial cardiopathy, coronary artery disease (CAD), pulmonary hypertension, and syncope.

## Heart failure

Several contributions focused on chronic HF and explored the potential risk factors that can impact clinical outcomes and mortality. Sleep apnea (SA) (including both obstructive and central SA) represents one of these factors, associated with a poor prognosis in patients with HF (1). In particular, in their retrospective observational study, Naito et al. demonstrated that in patients with HF, SA, which was not effectively suppressed by continuous positive airway pressure (CPAP), was associated with worse prognosis as compared to those with suppressed SA by CPAP. Another interesting risk factor is represented by the epigenetics, that can influence myocardial structure modifications during HF natural history (2). In this view, Han et al. provided evidence on the role of methyltransferase-like 5 (METTL5) whose loss in the animal model could promote pressure overload-induced cardiomyocyte hypertrophy and adverse remodelling. In their work, authors demonstrated how a missing m6A catalysation of 18S rRNA about the METTL5 would reduce the efficiency of mRNA translation of SUZ12, a core component of PRC2 complex, strong inhibitor of cardiac hypertrophy–related genes in hypertrophy hearts (3). One of this gene is GATA4. According to the study by Yan et al., GATA4 protein directly binds the promotor site of Cardiac ISL1-Interacting Protein (CIP), responsible to the production of CIP protein, that inhibits cardiac remodelling and protects the heart from HF after cardiac hypertrophy through IGF, mTORC2 and TGFβ signalling pathways, which regulate cardiac hypertrophy–related genes expression. A deeper understanding of cardiac remodelling is crucial in identifying potential molecular targets for pharmacotherapies. Furthermore, it is equally intriguing to anticipate the risk of mortality and re-hospitalization in patients with HF (4). Zhao et al. developed machine learning-based models specifically designed for patients with mild-reduced ejection fraction (HFmrEF), which demonstrated superior performance compared to traditional models in predicting mortality and rehospitalization. From the perspective of heart failure, an intriguing aspect is heart failure with preserved ejection fraction, where diastolic dysfunction of the left ventricle (LVDD) assumes a significant role. In septic patients LVDD is common and associated with high mortality. The endothelial dysfunction, characteristic of sepsis, results in increased vascular permeability, potentially causing pulmonary edema. Unfortunately, the quantification of acute pulmonary edema in septic patients using the lung ultrasound score (LUSS) is frequently challenging or unreliable. Indeed, Kahl et al. in a prospective cohort study observed that in 54 patients with sepsis, LVDD was not significantly associated with LUSS, even in the presence of severe pulmonary edema. Heart failure is also burdened by an important arrhythmic risk. Ventricular tachycardia, which occurs primarily in the context of structurally abnormal hearts, can result in severe hemodynamic compromise. Ventricular tachycardia ablation (VTA) is often a challenge. This procedure can be performed safely in selected high-risk patients using veno-arterial extracorporeal membrane oxygenation (VA-ECMO) support. In this regard, Sabbag et al. evaluated how early decanullation from VA-ECMO is associated with a higher survival at one year after VTA than in those in which it is not performed. Therefore, this procedure should be considered and performed immediately upon completion of VTA in most cases.

## Atrial cardiopathy

Within this Research Topic, several intriguing articles delve into the role of atrial cardiopathy as a significant substrate for promoting cardiac arrhythmias, including atrial fibrillation, as well as the pro-thrombotic state, which can occur independently of atrial fibrillation (5). In their review article, Donniacuo et al. provided valuable insights into the pathogenesis of COVID-19-related atrial fibrillation events, specifically addressing the cardiovascular safety profile of drugs used for the treatment of COVID-19. The authors highlighted multiple putative mechanisms, such as a reduced availability of angiotensin-converting enzyme 2, binding of viral spike protein to CD147 or sialic acid, enhancement of inflammatory signalling culminating in cytokine storm, endothelial damage, and increased adrenergic drive. Atrial cardiopathy, along with the intricate interplay of multiple mechanisms, may also contribute to the pathogenesis of many ischemic strokes of undetermined causes (6), especially embolic strokes of undetermined source (ESUS), where inflammation can promote both atrial fibrillation events and a prothrombotic state (7). In line with this perspective, the study conducted by Acampa et al. demonstrated that the presence of atrial cardiopathy, as assessed through electrocardiographic and echocardiographic markers, could serve as the underlying pathogenic mechanism in a subgroup of ESUS patients. Notably, this subgroup exhibits more severe neurological deficits and presents a clinical pattern resembling cardioembolic strokes attributed to atrial fibrillation. As regards the complications of stroke, spasticity stands out as one of them. In a randomized double-blind, placebo-controlled crossover study, Rosa et al. investigated the impact of nabiximols, a cannabinoid-derived drug, on post-stroke spasticity. The study findings indicate that nabixomols effectively alleviate spasticity without causing significant changes in blood pressure, heart rate, or cardiovascular complications in patients who in patients who have experienced a cerebrovascular accident. However, additional research is needed to explore other potential cardiovascular benefits of cannabinoids, such as their potential role in delaying the progression of atherosclerosis and inflammation.

## Coronary artery disease

The risk of mortality due to cardiac infarction can be enhanced in presence of complication related to the natural history of the disease, such as infections (8) in hospitalized patient and cardiac autonomic imbalance. Considering these concerns, prevention has become one of the top priorities in healthcare, to better improve the prognosis in patient with acute coronary syndrome (ACS). Given that, Liu et al. developed a 24-point risk score to use in ST-segment elevation myocardial infarction (STEMI), including seven variables such as age, Killip classification, insulin use, white blood cell count, serum albumin, diuretic use, and transfemoral approach. The score not only established a simple bedside tool to estimate the risk of developing infection for patient with STEMI but also demonstrated good performance for in-hospital all-cause death, and major adverse cardiovascular events (MACE) among these patients and even in the non-ST-elevation acute coronary syndrome (NSTE-ACS) treated with PCI. On the other hand, Duan et al. evaluated a possible extension of post-discharge GRACE score, considering in addition cardiac autonomic nerve imbalance, measured through the value of 24 h deceleration capacity (DC), a feasible and non-invasive indicator that captures autonomic activity-related modulations of heart rate. Combination of DC and the post-discharge GRACE score significantly enhanced the discriminatory ability and accuracy in the prediction of poor long-term follow-up prognosis. As regards the mortality induced by acute myocardial infarction, it is necessary to consider ischemia reperfusion injury (IRI), a possible cause of secondary myocardial damage. Evaluating the possible therapeutic strategies able to attenuate the IRI, Wei et al. analysed the protective role of Danlou tablet (Dan), a Chinese herbal compound. This study provided for the first time evidence that Dan could attenuate cardiomyocyte apoptosis and ischemia-reperfusion injury, by experiments conducted *in vivo*, using an acute IRI model in mice, and *in vitro*, through oxygen-glucose deprivation-reperfusion (OGD/R)-induced apoptosis in primary neonatal rat cardiomyocytes (NRCMs). Mechanistically, Dan could activate proliferator-activated receptor gamma (PPAR-γ) in both models, while inhibition of PPAR-γ could attenuate the protective effect of Dan. These data provide a new potential strategy for the precise treatment of ischemic heart diseases complications. Left ventricular (LV) remodelling is one of the possible functional complications following a STEMI. Pharmacological interventions, able to prevent LV remodelling following a STEMI, improve the outcome of this condition. Egea Iborra et al. evaluated the possible effects of paroxetine, a GRK2 inhibitor also known as beta-adrenergic receptor kinasi 1. Among the drugs used for neuronormal antagonism (beta-blockers,angiotensin converting enzyme inhibitors, mineralcorticoid receptor antagonists, angiotensin receptor blockers), capable of preventing LV remodelling, only beta-blockers act directly on GRK2 as paroxetine. For this reason, paroxetine, sharing the same molecular target with beta-blockers, could be used when these are contraindicated (for example in subjects with hypotension) or poorly tolerated.

## Pulmonary hypertension

Another topic of particular interest in the management of cardiovascular patient is pulmonary hypertension, which is often a consequence of heart disease. In this context, given the high impact of this condition on cardiovascular health, Miao et al. presented a more in-depth insight about the complex molecular mechanisms of chronic thromboembolic pulmonary hypertension, highlighting the role of specific mRNAs, miRNAs, and circRNAs and inflammatory cells recruitment in the progression of the disease. Instead, Rodrigues et al. evaluated the impact of a blunted cardiac autonomic modulation and a pro-inflammatory profile on pulmonary artery pressure (PAPs) in systemic sclerosis patients, suggesting a relationship among cardiac autonomic control, inflammatory status, and cardiopulmonary mechanics.

## Syncope

A common challenge in cardiovascular medicine involves the impact of syncope in childhood, which exhibits a high prevalence and multiple etiologies (9). To distinguish syncope due to an excess in vasovagal reflex [vasovagal (VVS) syncope] and syncope due to a conversion disorder [psychogenic (PPS)] poses a significant difficulty, as these two forms share several clinical manifestations, including repeated episodes of transient loss of consciousness and falls usually without convulsions. On this argument, Li et al. designed a study to evaluate a clinical manifestation-based scoring, consisting of 4 variables, aiming to aid in the initial differential diagnosis between PPS and VVS. On a different note, to gain a better understanding of the pathophysiology of VVS, Wang et al. investigated the profile of plasma human growth cytokines. Their findings revealed that elevated plasma concentrations of HGF and IGFBP-1, and decreased EGF were typical in pediatric VVS.

*In conclusion*, the remarkable contributions within this Research Topic have greatly advanced our comprehension of various aspects within cardiovascular medicine. These studies have illuminated intricate physiological and pathogenic mechanisms, which bear significant clinical implications for patient management. Moreover, they have provided valuable insights and recommendations that pave the way for further exploration in this rapidly evolving field.
